# Functional characterization of the *ZEB2* regulatory landscape

**DOI:** 10.1093/hmg/ddy440

**Published:** 2018-12-26

**Authors:** Reut Bar Yaacov, Reut Eshel, Einan Farhi, Fania Shemuluvich, Tommy Kaplan, Ramon Y Birnbaum

**Affiliations:** 1 Department of Life Sciences, Ben-Gurion University of the Negev, Beer-Sheva, Israel; 2 Center of Evolutionary Genomics and Medicine, Ben-Gurion University of the Negev, Beer-Sheva, Israel; 3 School of Computer Science and Engineering, Hebrew University of Jerusalem, Jerusalem, Israel

## Abstract

Zinc finger E-box–binding homeobox 2 (*ZEB2*) is a key developmental regulator of the central nervous system (CNS). Although the transcriptional regulation of *ZEB2* is essential for CNS development, the elements that regulate *ZEB2* expression have yet to be identified. Here, we identified a proximal regulatory region of *ZEB2* and characterized transcriptional enhancers during neuronal development. Using chromatin immunoprecipitation sequencing for active (H3K27ac) and repressed (H3K27me3) chromatin regions in human neuronal progenitors, combined with an *in vivo* zebrafish enhancer assay, we functionally characterized 18 candidate enhancers in the *ZEB2* locus. Eight enhancers drove expression patterns that were specific to distinct mid/hindbrain regions (*ZEB2#e3* and *5*), trigeminal-like ganglia (*ZEB2#e6* and *7*), notochord (*ZEB2#e2*, *4* and *12*) and whole brain (*ZEB2#e14*). We further dissected the minimal sequences that drive enhancer-specific activity in the mid/hindbrain and notochord. Using a reporter assay in human cells, we showed an increased activity of the minimal notochord enhancer *ZEB2#e2* in response to *AP-1* and *DLX1*/*2* expressions, while repressed activity of this enhancer was seen in response to *ZEB2* and *TFAP2* expressions. We showed that Dlx1 but not Zeb2 and Tfap2 occupies *Zeb2#e2* enhancer sequence in the mouse notochord at embryonic day 11.5. Using CRISPR/Cas9 genome editing, we deleted the *ZEB2#e2* region, leading to reduction of *ZEB2* expression in human cells. We thus characterized distal transcriptional enhancers and trans-acting elements that govern regulation of *ZEB2* expression during neuronal development. These findings pave the path toward future analysis of the role of *ZEB2* regulatory elements in neurodevelopmental disorders, such as Mowat–Wilson syndrome.

## Introduction

Zinc finger E-box–binding homeobox (ZEB2) is a key transcription factor that acts as a multifunctional regulator during nervous system development. ZEB2 contains two zinc finger domains and a homeodomain-like sequence (1) and interacts with the TGF-ß superfamily signaling regulators, Smads, to regulate the expression of their downstream genes (1,2). *ZEB2* is expressed in the developing neural tube, as well as in neural crest cells, the hippocampus and the cerebral cortex (3,4). *Zeb2* knockout mice die around embryonic day (E) 9.5 and exhibit severe neural plate and neural crest defects from E8.5 (4,5). Conditional *Zeb2* knockout mice show open neural tube defects, delamination arrest of neural crest cells (4) and hypo-cellularity of enteric neurons, all of which are characteristic of Mowat–Wilson syndrome (MWS) (6). MWS is a neurodevelopmental syndrome characterized by a combination of defects with variable penetrance, including seizures, that can be caused by *de novo* mutations in *ZEB2* (7). While more than 100 heterozygous *ZEB2* haploinsufficiency mutations have been demonstrated in MWS, some phenotypic cases do not present *ZEB2*-coding region mutations, suggesting other genomic variants—perhaps in *ZEB2* regulatory elements—might underlie the molecular basis of this syndrome (8).

The presence of Zeb2 throughout the nervous system development highlights its vital regulatory role in this process (9). For instance, Zeb2 regulates the development of neural progenitors in the subpallium, which gives rise to both cortical and striatal interneurons. Conditional knockout of *Zeb2* predominantly in the mouse medial ganglionic eminence (MGE) results in a decrease in the number of cortical GABAergic interneurons and a concomitant increase in the number of striatal GABAergic interneurons (10), indicating that Zeb2 constitutes a switch of inhibitory interneurons that are essential for brain development. The expression of *Zeb2* is directly regulated by homeobox transcription factors Dlx1 and Dlx2, which are essential for subpallial development (10–12). Moreover, Zeb2 represses the expression of Nkx2.1, a transcription factor in the MGE that induces the production of cortical GABAergic interneurons and represses the production of striatal GABAergic interneurons, indicating that Zeb2 promotes specification of cortical GABAergic interneurons via Nkx2.1 regulation (10,13). Thus, the spatiotemporal expression of *Zeb2* is essential for interneuron production, migration and differentiation during brain development.

The spatiotemporal regulation of *ZEB2* is complex and involves various regulatory elements, including alternative promoters and specific enhancers that contribute to the multifactorial function of this transcription factor (10,14,15,16). In humans, *ZEB2* has two known promoters, 1a and 1b, that are followed by distinct non-coding first exons located 2.2 kb apart that are spliced to a common exon 2 containing the translation initiation site (16). The transcription factor AP-1, when activated by TNFα in cancer cells, binds to a specific site in promoter 1b that activates *ZEB2* transcription (16). In addition, hypermethylation of the *Zeb2* promoter was found to repress its expression (17). Recently, it was suggested that *Zeb2* expression is regulated not only by the promoter but also by a number of enhancers located distally from the *Zeb2* promoters (10,15). A kidney-specific enhancer located 1.2 Mb from the *Zeb2* promoter is active in neonatal rats and contributes to *Zeb2* expression in renal tissue (15). Furthermore, two distal enhancers activated by Dlx2 in the developing subpallium were suggested to control *Zeb2* expression in mouse E11.5 and E13.5 embryos (10).

At the same time, transcriptional enhancers that require physical interactions with the targeted promoters are also dependent on the 3D structure of the genome and the topologically associating domains (TADs) (18). TADs consist of continuous genomic regions that preferentially form contacts within themselves to a higher degree than with neighboring regions in the context of compacted chromatin in the nucleus (19–21). During neuronal differentiation, novel TAD boundaries appear in close proximity to the promoters of developmentally regulated genes through the involvement of enhancer–promoter interactions and specific transcription factors (TFs) that contribute to creating sub-TADs that are required for transcriptional regulation, as recently reported (22). However, it remains unclear how neuronal enhancers regulate the expression of *ZEB2* during development of the nervous system.

In this study, we determined chromatin organization at the *ZEB2* locus and assessed the generation of sub-TADs in differentiated neurons, defining the *ZEB2* regulatory region. We further identified and characterized neuronal enhancers in the *ZEB2* locus that regulate its tissue-specific expression during nervous system development. The temporal and spatial regulations of these enhancers likely contribute to the multiple roles of ZEB2, with their deregulation possibly leading to pathogenic processes.

## Results

### Chromatin interactions are enriched at the *ZEB2* locus in neuronal tissues

**Figure 1:**
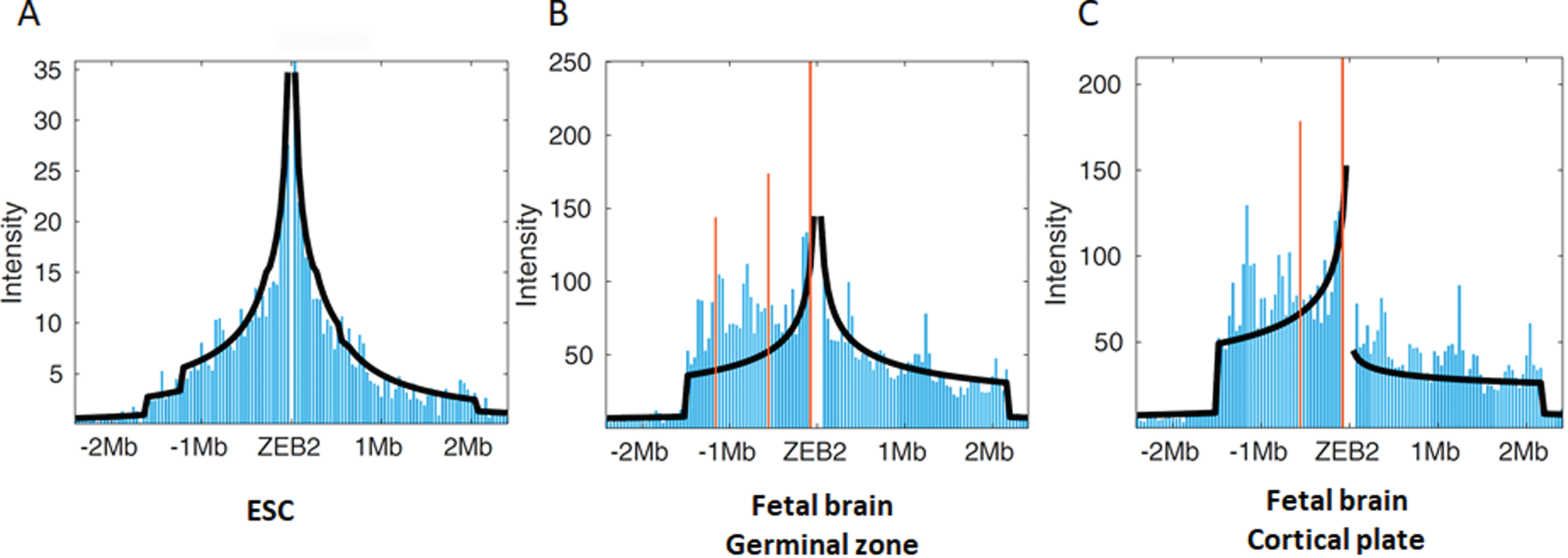
Analysis of TAD boundaries in the *ZEB2* locus. Hi-C data analysis of the *ZEB2* locus by PSYCHIC in (**A**) H1-ESC, (**B**) the germinal zone of human fetal brain and (**C**) the cortical plate of human fetal brain. Each column represents interaction intensity in a 50 kb window. Orange columns represent a region of significant interaction with the TAD boundary (False positive rate (FDR) <0.01).

To determine the regulatory region that modulates *ZEB2* neuronal expression, we initially examined the organization of the genome into TADs in the *ZEB2* locus. Since TAD boundaries are typically conserved across different cell types (19), we reasoned that no changes in TAD boundaries likely occur in the *ZEB2* locus during neuronal differentiation. Using PSYCHIC (23), a computational approach for analyzing Hi-C data and identifying promoter–enhancer interactions, we analyzed chromatin interactions from Hi-C data collected from various cell/tissue types (19,20,24,25). We found that in the H1 human embryonic stem cell line (H1-ESC), where *ZEB2* is not expressed, the TAD boundaries are located far from the gene (>1.5 Mb). In contrast, in the germinal zone and cortical plate of human fetal brain, a sub-TAD boundary (chr2: 145,280,000-145,300,000; hg19) is located in close proximity to *ZEB2* transcription start site (chr2: 145,277,958) and promoter region, indicating that the ZEB2 regulatory elements are located in the same sub-TAD (Fig. 1; Supplementary Material, Fig. S1) (25). In human fetal brain, the proximal region of the *ZEB2* TAD boundary that spans ~80 Kb displays a significantly high chromatin interaction frequency with the *ZEB2* promoter region both in the germinal zone and in the cortical plate but not in H1-ESC, suggesting that the regulatory elements of *ZEB2* are located in the proximal region of this sub-TAD boundary. Indeed, the proximal region of this sub-TAD boundary is enriched in epigenetic marks of active enhancers (H3K4me1 and H3K27ac) in human brain tissues but not in H1-ESC, suggesting that this *ZEB2* TAD contains clusters of regulatory elements that are required for neuronal activity. Thus, we used the *ZEB2* TAD and the extensive neuronal enhancer marks detected to define the potential *ZEB2* regulatory region involved in human brain development.

### *ZEB2* enhancer candidates in the developing central nervous system

To identify neuronal *ZEB2* enhancers that are active during development, we analyzed enhancer-associated chromatin immunoprecipitation sequencing (ChIP-seq) data sets in different human neuronal cells, focusing on the *ZEB2* locus (26,27) (see Materials and Methods). As *ZEB2* is expressed in MGE-like progenitors during brain development (10), we analyzed ChIP-seq data from human MGE-like progenitors differentiated from H9-ESC (Eshel *et al.*, unpublished). The inhibitory interneuron differentiation procedure follows four major developmental stages that include the appearance of human stem cells (H9-hESCs), MGE-like progenitors (day 26), differentiated GABAergic-like interneurons (day 39) and somatostatin-enriched GABAergic interneurons (day 55). We initially tested *ZEB2* expression during the four stages of interneuron differentiation and found that ZEB2 was mostly absent in H9-ESCs but was highly expressed in MGE-like progenitors, supporting the hypothesis that ZEB2 acts as a developmental switch, steering progenitor cells to become cortical or striatal interneurons (Supplementary Material, Fig. S2A). Using ChIP-seq for H3K27ac and H3K27me3 on MGE-like progenitors, we focused our analysis on the *ZEB2* locus and selected candidate sequences that present an active enhancer histone mark (H3K27ac) but lack the repressive histone mark (H3K27me3). Using publicly available ChIP-seq data (27), these candidates progenitor enhancers were also analyzed for the presence of active enhancer marks (H3K27ac and H3Kme1) in neuronal progenitors and fetal brain (male and female). We thus identified 18 enhancer candidates in the *ZEB2* locus, with 3 candidates being intergenic, 14 being intronic and 1 being an exonic sequence (Supplementary Material, Table S1). Together, these 18 candidates might participate in transcription regulation of *ZEB2* during development of the central nervous system (CNS).

### *In vivo* activity of neuronal enhancers at the *ZEB2* locus

**Figure 2:**
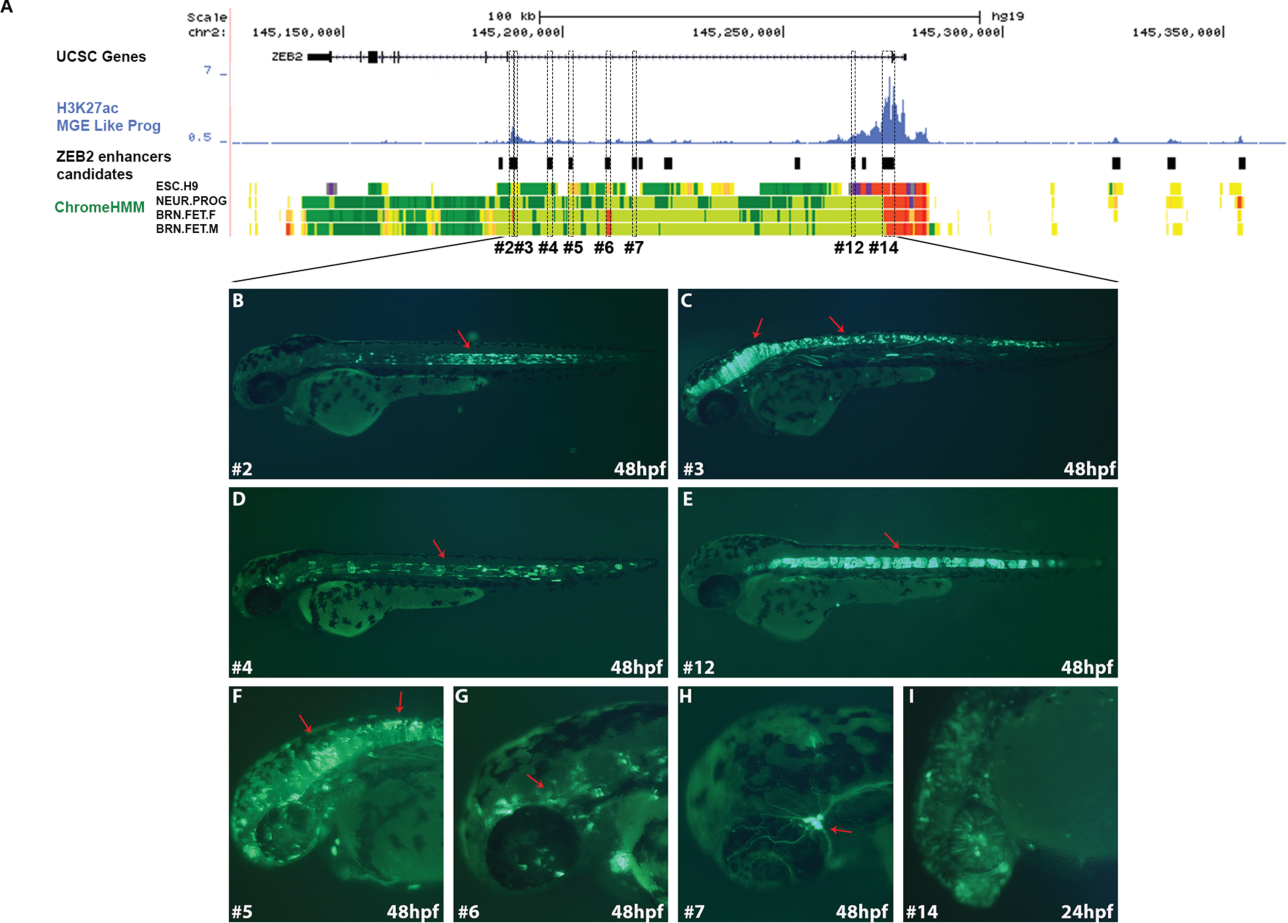
*In vivo* functional enhancers in the *ZEB2* locus. (**A**) UCSC Genome Browser (http://genome.ucsc.edu) tracks represent UCSC genes, H3K27ac ChIP-seq peaks of human interneuron progenitors, selected enhancer candidates and chrome ImputedHMM track from roadmap displays chromatin state segmentation for H1 and H9 ESC-derived neurons and human fetal brain (female and male). Black boxes represent enhancer candidates that were tested in zebrafish. Dashed rectangles represent positive enhancers in zebrafish. (**B–J**) Tissue-specific enhancers in zebrafish embryos at 24 and 48 hpf reflected by GFP expression in the (B) notochord (*ZEB2#e2*), (C) mid/hindbrain and spinal cord (*ZEB2#e3*), (D and E) notochord (*ZEB2#e4* and *12*), (F) mid/hindbrain and spinal cord (*ZEB2#e5*), (G and H) specific neurons with morphology-like trigeminal ganglia (*ZEB2#e6* and *7*) and (I) CNS (*ZEB2#e14*).

To determine the *in vivo* activity of the 18 enhancer candidates during development, we tested their activities using an enhancer assay in zebrafish. Zebrafish enhancer assays are rapid and efficient method to determine spatiotemporal enhancer activity in real time during development. Indeed, human and mouse enhancer sequences have been successfully characterized in zebrafish, regardless of the extent of their conservation in the zebrafish genome (28–30). The enhancer candidates were thus cloned into a zebrafish enhancer vector containing the E1b minimal promoter followed by the green fluorescent protein (GFP) reporter gene (31). The vectors were microinjected into one-cell-stage zebrafish embryos along with the *Tol2* transposase for genomic integration (32). GFP activity was monitored 24, 48 and 72 h post-fertilization (hpf) and compared to the known *ZEB2* expression pattern (33) (Supplementary Material, Fig. S2B). Nine candidates drove GFP expression in specific tissues (Supplementary Material, Table S2). *ZEB2#e3* and *5* drove GFP expression in the mid/hindbrain and spinal cord; *ZEB2#e14* drove GFP expression in the CNS; two enhancers *ZEB2#e6* and *7* drove GFP expression in specific neurons with trigeminal ganglia-like morphology; and three enhancers *ZEB2#e2*, *4* and *12* drove GFP expression in the notochord (Fig. 2; Supplementary Material, Figs S3 and S4). Furthermore, *ZEB2#e2* and *3* together were found to drive a reporter gene in the basal root ganglia and mid/hindbrains of embryonic day 11.5 mice (34) that resembled their activity in our zebrafish experiments. Overall, we characterized eight positive neuronal enhancers in zebrafish that could regulate *ZEB2* expression during brain development. In addition, the *ZEB2#e13* enhancer drove GFP expression in somitic muscles (Supplementary Material, Fig. S4).

### Minimal enhancer sequences that are not necessarily evolutionary conserved are sufficient to drive tissue-specific activity

To define the minimal sequence required for the tissue-specific activity of the identified enhancers, we carried out a series of segmental analyses of the enhancer sequences using a zebrafish enhancer assay. To this end, we selected the mid/hindbrain (*ZEB2#e5*) and notochord (*ZEB2#e2*, *4* and *12*) enhancers (Fig. 2B and D–F). We hypothesized that although enhancer sequences that drive similar expression patterns are not identical, they should share transcription factor-binding sites (TFBS) that are required for their activity. Therefore, we dissected these sequences to characterize the minimal enhancer sequence using a zebrafish enhancer assay.

*ZEB2#e5*, showing hindbrain, midbrain and spinal cord expressions (Supplementary Material, Fig. S5), was divided into three segments, with segments 1 and 3 being evolutionary conserved to zebrafish (35) (Supplementary Material, Fig. S5A). Segments 1 and 2 did not drive GFP expression in either the mid/hindbrain or spinal cord (Supplementary Material, Fig. S5B and C), although segment 3 showed high and specific expression in both mid/hindbrain and spinal cord (Supplementary Material, Fig. S5B and C). We also tested for the activity driven by segments 2 and 3 joined together and identified similar expression as driven by segment 3 alone, thus recapitulating the expression pattern seen with the full-length enhancer. We further dissected the conserved segment 3 sequence and showed that the zebrafish-conserved sequence drove a similar expression pattern as did the human sequence (Supplementary Material, Fig. S5C). Thus, the evolutionarily conserved region spanning segment 3 likely serves as the minimal enhancer required for mid/hindbrain activity.

**Figure 3:**
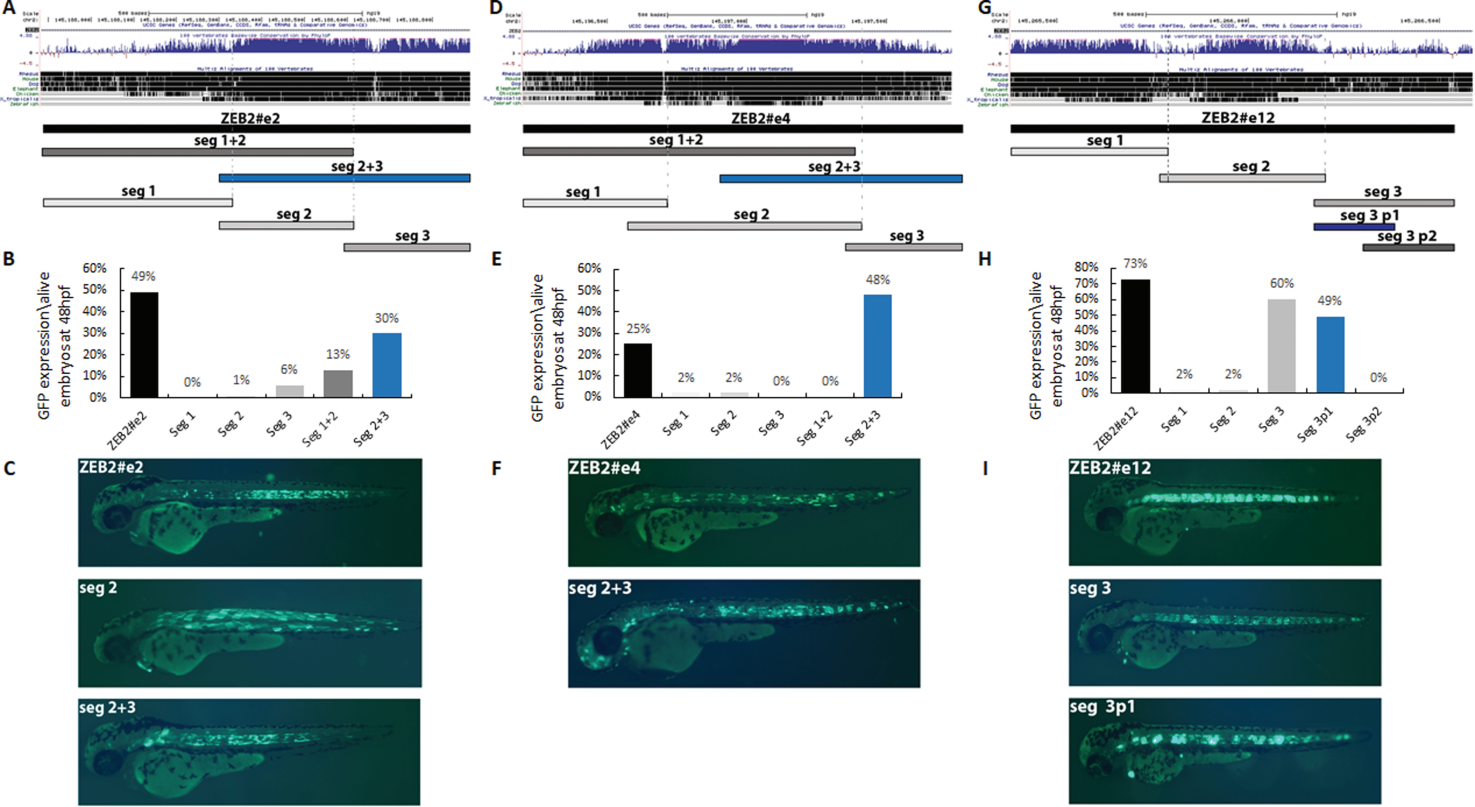
Segmental analysis of the three notochord enhancers: *ZEB2#e2*, *4* and *12*, in zebrafish embryo at 48 hpf. (**A–C**) Segmental analysis of *ZEB2#e2* that was divided into three overlapping segments (segments 1–3). (A) UCSC Genome Browser conservation track (http://genome.ucsc.edu). (B) A graph displaying the number of embryos showing GFP expression in the notochord out of all live embryos at 48 hpf. (C) Zebrafish enhancer assays for *ZEB2#e2* segments show notochord GFP expression (and in somitic muscles) for the entire ChIP-seq peak, somitic muscle GFP expression for segment 2 and notochord and somitic muscle GFP expression for segment 2+3 (no GFP expression for segments 1, 3 or 1+2). (**D–F**) Segmental analysis of *ZEB2#e4* that was divided into three overlapping segments (segments 1–3). (D) UCSC Genome Browser conservation track (http://genome.ucsc.edu). (E) A graph displaying the number of embryos showing GFP expression in the notochord out of live embryos at 48 hpf. (F) Zebrafish enhancer assays for *ZEB2#e4* segments show notochord GFP expression for the entire ChIP-seq peak and notochord GFP expression with segment 2+3 (no GFP expression with segments 1, 2, 3 and 1+2). **(G–I)** Segmental analysis of *ZEB2#e12* that was divided into three overlapping segments (segments 1–3), with segment 3 being further divided into additional two parts (3p1 and 3p2). (G) UCSC Genome Browser conservation track (http://genome.ucsc.edu). (H) A graph displaying the number of embryos showing GFP expression in the notochord out of all live embryos at 48 hpf. (I) Zebrafish enhancer assays for *ZEB2#e12* segments show notochord GFP expression for the entire ChIP-seq peak and notochord GFP expression with segments 3 and 3p1 (no GFP expression with segments 1, 2, 3 and 3p2).

*ZEB2#e2*, showing high notochord and somatic muscle enhancer activity (Fig. 2B), was also divided into three segments, based on the level of evolutionary conservation (35) (Fig. 3A). Segments 1 and 3 did not drive GFP expression in either the notochord or somatic muscles (Fig. 3B and C). Segment 2 showed activity in somatic muscles but not in the notochord (Fig. 3B and C). Next, we tested segments 2 and 3 joined together and noted strong activity in both the notochord and somatic muscles, recapitulating the expression pattern seen with the full-length enhancer. Furthermore, segments 1 and 2, when together, showed strong activity in somatic muscles which correlated with segment 2 activity (Fig. 3B and C). Thus, our results demonstrated that the evolutionarily conserved region spanning segments 2 and 3 could serve as the minimal enhancer necessary for notochord and somatic muscle enhancer activity.

*ZEB2#e4*, showing strong activity in the notochord (Fig. 2D), was likewise divided into three segments based on the level of evolutionary conservation (Fig. 3D). We found that none of the three segments alone was active in the notochord (Fig. 3E and F). However, when segments 2 and 3 were tested together, strong activity in the notochord was observed, recapitulating the activity of the full-length enhancer (Fig. 3E and F). It thus seems that segments 2 and 3 together play an important role in *ZEB2#e4* activation in the notochord.

Finally, *ZEB2#e12*, which also showed strong activity in the notochord and somatic muscles (Fig. 2E), was analyzed. After dividing this enhancer into three segments (Fig. 3G), we found that segment 1 did not drive enhancer activity. However, segment 2 was active in somitic muscles and segment 3 showed strong activity in the notochord (Fig. 3H and I). Moreover, when segments 1 and 2 were tested together, we observed GFP expression in the somatic muscles (Fig. 3H and I). When segments 2 and 3 were tested together, we observed strong activity in the notochord and somitic muscles (Fig. 3H and I). We, therefore, tested whether segment 3 includes the minimal notochord enhancer, by dividing segment 3 into two additional parts (p1 and p2). Segment 3p1 drove strong notochord GFP expression, while segment 3p2 caused no such expression. Thus, our results demonstrated that segment 3p1, which is not evolutionarily conserved, is likely the minimal enhancer within *ZEB2#e12*.

In summary, these findings suggest that minimal enhancer sequences that are not necessarily evolutionarily conserved can drive tissue-specific activity.

### *ZEB2#e2* activity is increased by DLX1/2 and decreased by ZEB2 and TFAP2

**Figure 4:**
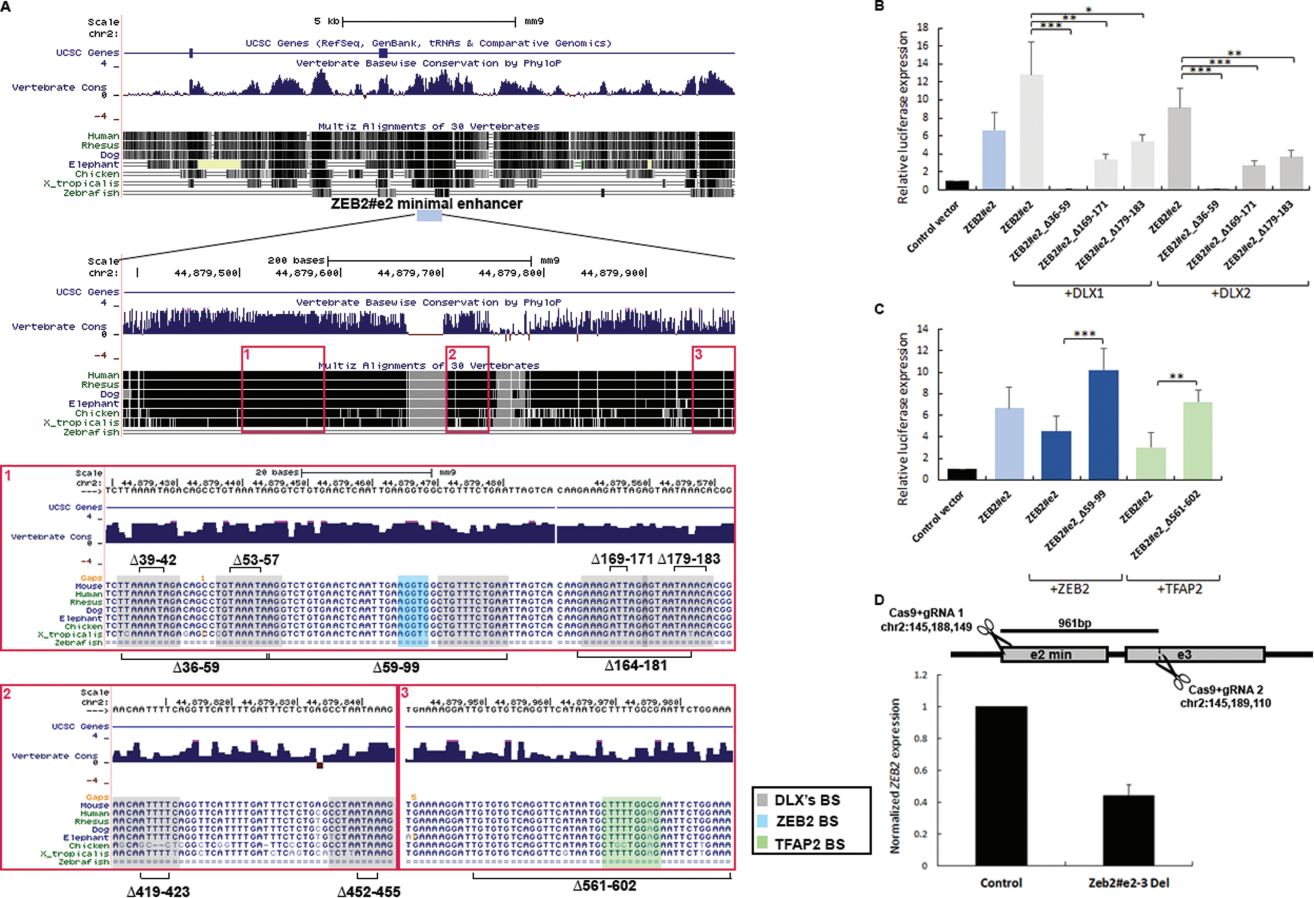
*ZEB2#e2* activity is increased by DLX1/2 and decreased by ZEB2 and TFAP2. (**A**) UCSC Genome Browser conservation track of the *ZEB2#e2* minimal sequence. Predicted TFBS for DLX1/2 (gray rectangle), ZEB2 (blue rectangle) and TFAP2 (green rectangle) are presented, along with the deletions of the binding sites for DLXs (∆39-42, ∆53-57, ∆59-99, ∆169-171, ∆179-183, ∆419-423 and ∆452-455), ZEB2 (∆59-99) and TFAP2 (∆561-602). (**B**) Luciferase assay demonstrating transcriptional activation mediated by the *ZEB2#e2* minimal enhancer sequence in HEK293 cells. The *ZEB2#e2* minimal enhancer showed 4–6-fold increased activity, as compared to the control (plasmid pGL4.23). DLX1/2 expression plasmids increased *ZEB2#e2* activity 10–15-fold, as compared to the control. *ZEB2#e2*_∆36-59 mutant did not drive the luciferase reporter gene, while *ZEB2#e2*_∆169-171 and *ZEB2#e2*_∆179-183 mutants decreased the luciferase activity 3–4 and 2–2.5-fold, respectively. (**C**) ZEB2 and TFAP2 (both TFAP2α and TFAP2γ) expression plasmids decreased *ZEB2#e2* activity 1.5–2.3-fold, as compared to the enhancer itself. ZEB2 expressed from the plasmid increased luciferase activity 2-fold in the *ZEB2#e2*_∆59-99. TFAP2 expressed from the plasmid increased luciferase activity 2.3-fold in the *ZEB2#e2*_∆561-602 mutant. Relative luciferase expression results are presented after normalization to Renilla activity and represent the mean ± standard deviation of three independent experiments (*P* < 0.05; Student’s independent *t*-test). (**D**) Predicted cleavage sites for paired guide RNA that was used for CRISPR/Cas9-mediated deletion of 961 bp. Quantitative qPCR shows reduced *ZEB2* expression upon heterozygous *ZEB23e2/3* deletion in HEK293 cells*.* Normalized expression levels of *ZEB2* relative to control (*P*-value; mean ± standard error; *n* = 4; Student’s *t*-test).

As the three notochord enhancers are similar in terms of their function but not in sequence, we aimed to identify the distinct transcription factors that are required for notochord *ZEB2* enhancer activity. Using JASPAR (36), we analyzed the sequences of the three positive notochord enhancers (*ZEB2#e2*, *4* and *12*) for predicted TFBSs. From the multiple predicted TFBSs (Supplementary Material, Table S4), we selected six TFs (DLX1/2, AP-1, ZEB2, TFAP2, FOXG1) that preferentially expressed in neuronal tissues and shared predicted binding sites between these enhancers. Therefore, these six TFs are the top candidates for regulating *ZEB2* expression in such tissues. DLX1/2 and AP-1 were previously reported as *ZEB2* expression activators (10,16), while ZEB2, TFAP2 and FOXG1 were suggested to serve as both repressors and activators (9,37–40). Next, as a first step to quantifying the effect of each transcription factor on enhancer activity, we performed a dual luciferase reporter assay in human embryonic kidney cells (HEK293) to determine TF-mediated effects on enhancer activity. Notably, HEK293 cells were chosen for these experiments because of their relative ease of use, especially since the activity of the enhancers was tested while co-introducing expression of the relevant TFs, thereby avoiding the endogenic proteins expressed in these cells. Specifically, we tested the activities of the three notochord enhancers in HEK293 cells, following by co-transfection with the relevant TF. Firstly, we showed that whereas the minimal *ZEB2#e4* and *12* sequences had no activity in the HEK293 cells, the minimal *ZEB2#e2* sequence showed a 7-fold elevation of reporter gene expression, as compared to the control vector (empty pGL4.23 vector). Next, we found that co-transfection with *FOXG1* yielded no significant enhancer activation. Secondly, co-transfection with *DLX1*, *DLX2*, *ZEB2*, *TFAP2* and *AP-1* significantly modulated the activity of the minimal *ZEB2#e2* enhancer (Fig. 4; Supplementary Material, Fig. S6), while only *TFAP2* modulated the activity of minimal *ZEB2#e4* enhancer (Supplementary Material, Fig. S6).

Co-transfection of *ZEB2#e2* with *ZEB2* or *TFAP2* led to 30% and 50% significant reductions in enhancer activity, respectively (*P* < 0.05; Student’s *t*-test; Fig. 4B and C). In contrast, co-transfection of *ZEB2#e2* with *DLX1*, *DLX2* or *AP-1* resulted in significant elevation of enhancer activity (*P* < 0.05, Student’s *t*-test; Fig. 4B; Supplementary Material, Fig. S6). Taken together, the data indicate that while *ZEB2* and *TFAP2* reduced *ZEB2#e2* activity, *DLX1* and *DLX2* elevated its activity. Moreover, although *ZEB2#e4* and *12* contain binding sites for these TFs, we found that only *TFAP2* activated *ZEB2#e4* and that none of the tested TFs modulated *ZEB2#e12* in our experimental system (Supplementary Material, Fig. S6).

To validate the effect of each TF on the minimal *ZEB2#e2* enhancer activity, we generated a series of deletions (mutations) in which the predicted TF-binding sites were deleted from the enhancer sequence and tested for the impacts of such deletions using a dual luciferase reporter assay (Fig. 4). In the *ZEB2#e2*_∆36-59 mutant, the two predicted sites of DLX1/2 binding were deleted (Fig. 4B). Co-transfection of the *ZEB2#e2*_∆36-59 mutant with or without DLX1/2 abolished enhancer activity. Interestingly, we tested the effects of deleting each DLX-binding site separately (∆39-42 and ∆53-57) and found that co-transfection of either *ZEB2#e2*_∆39-42 and *ZEB2#e2*_∆53-57 with the *DLX* variants did not disrupt enhancer activity (Supplementary Material, Fig. S6D), suggesting that these two DLX-binding sites serve redundant functions in activating *ZEB2#e2*.

In the *ZEB2#e2*_∆59-99 mutant, the binding sites for DLXs, ZEB2 and AP-1 were deleted. While co-transfection of the ∆59-99 mutant with *DLX1/2* and *AP-1* showed no change in enhancer activity (data not shown), co-transfection with *ZEB2* resulted in a doubling of enhancer activity (Fig. 4C).

In the *ZEB2#e2*_∆164-181 mutant, two DLX-binding sites were deleted (169-171, 179-183). Co-transfection of the ∆164-181 mutant with *DLX1/2* resulted in a 70–85% reduction in enhancer activity (Supplementary Material, Fig. S6C). We generated two additional deletions affecting each DLX-binding site (∆169-171 and ∆179-183) and found that when both mutants were co-transfected along with *DLX1/2*, a significant reduction (70–75%) in enhancer activity was observed (Fig. 4B). In contrast, other DLX-binding site mutants (∆419-423 and ∆452-455) had no significant impact on enhancer activity when co-transfected with any of the *DLX*s (data not shown). Thus, DLX-binding sites are likely required for enhancer activity, although the different sites do not have the same regulatory impact.

In *ZEB2#e2*_∆561-602 mutant, the binding site of TFAP2 was deleted (Fig. 4C). Co-transfection of *ZEB2#e2*_∆561-602 with *TFAP2* showed elevation of enhancer activity, suggesting a repressor effect of *TFAP2* on this enhancer (Fig. 4C). Thus, the activity of *ZEB2#e2* is elevated by *DLX1/2* and is reduced by *ZEB2* and *TFAP2* in HEK293 cells.

Finally, we tested the *in vivo* effect of mutated DLX1/2-binding sites (∆36-59 and ∆164-181) on *ZEB2#e2* enhancer activity using a zebrafish enhancer assay. Zebrafish embryos were micro-injected with enhancer vectors containing the *ZEB2#e2* sequence mutated for DLX1/2-binding sites. A significant decrease in embryos expressing GFP in the notochord was noted (14% and 19%), as compared to embryos micro-injected with a control enhancer sequence (40%; *P* < 0.05; chi square test). These results demonstrate the importance of DLX1/2-binding sites for the activity of this notochord enhancer (Supplementary Material, Table S2). Furthermore, zebrafish embryos that were micro-injected with enhancer vectors containing the *ZEB2#e2* sequence mutated for ZEB2 or TFAP2 binding sites showed similar numbers of embryos expressing GFP in the notochord (32% and 34%, respectively), as compared to embryos micro-injected with a control enhancer sequence (Supplementary Material, Table S2).

### Functional TFBS for *ZEB2#e2* lacks nucleotide variation in human population

To evaluate the potential functional effect of genetic variation on the characterized enhancers, we screened our enhancer sequences for single nucleotide polymorphisms (SNPs) in the human population. This screen revealed that common SNPs (i.e. with minor allele frequency (MAF) >1%) were found within the enhancer sequences of *ZEB2* locus, including *ZEB2#e2*. However, upon examining the TFBSs within *ZEB2#e2*, we found no common SNPs in DLX1/2-, ZEB2- or TFAP1-binding sites that proved to be functional in our reporter assay (Fig. 4). Furthermore, none of these TFBSs present any rare nucleotide variants, except for the first *ZEB2#e2* DLX1/2-binding site (Supplementary Material, Fig. S7). Deletion of this first DLX1/2-binding site (∆39-42) did not disrupt the enhancer activity (Supplementary Material, Fig. S6D), but deletion of this binding site along with a nearby DLX1/2- binding site completely abolished the enhancer activity (∆53-57), indicating redundancy of these two DLX1/2 binding sites. These results suggest that the identified TFBS in *ZEB2#e2* might be under negative selection, likely to preserve their functionality.

**Figure 5:**
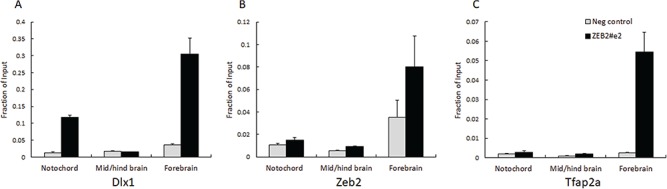
Zeb2 and Tfap2α proteins present on *Zeb2#e2* in mouse E11.5 neuronal tissues. ChIP-qPCR analysis with (**A**) anti-Dlx1, (**B**) Zeb2 and (**C**) Tfap2**α** antibodies on *ZEB2#e2* region in notochord, mid/hindbrain and forebrain. Fold enrichment is presented as a fraction of input. The control region represents a target sequence without potential binding sites for Dlx1, Zeb2 or Tfap2α.

### Deletion of enhancer elements alters *ZEB2* expression

To further examine the function of *ZEB2#e2* enhancer, we performed CRISPR/Cas9 gene editing to delete the endogenous element in HEK293. We used several pairs of gRNA and have successfully generated heterozygous deletion mutant that removed 961 bp (chr2:145188149-145189110, hg19), encompassing *ZEB2#e2* and 3 enhancers (Fig. 4D). As *ZEB2#e2* drove reporter expression in HEK293, we preformed quantitative PCR (qPCR) to examine the effect of the deletion on *ZEB2* expression levels. We found ~50% reduction of *ZEB2* expression in the mutant cells compared to control cells (*n* = 4; Fig. 4D). As the deletion contains both *ZEB2#e2* and part of *ZEB2#e3*, loss of DNA elements that function as enhancers reduced ZEB2 expression. Thus, our results suggest that deletion of *ZEB2#e2* and *3* enhancers might lead to ZEB2 loss of function.

### Dlx1, Zeb2 and Tfap2a proteins are detected on *Zeb2#e2* enhancer *in vivo*

The effects of Dlx1, Zeb2 and Tfap2 on *Zeb2#e2* enhancer activity in the reporter assay suggest *in vivo* roles for these TFs in terms of Zeb2 transcription regulation. Therefore, we investigated the possibility that Dlx1, Zeb2 and Tfap2 are transcriptional targets of *Zeb2#e2*. As *Zeb2#e2* is an active enhancer in the notochord, we performed ChIP followed by qPCR (ChIP-qPCR) on mouse E11.5 notochord, mid/hindbrain and forebrain tissues using anti-Dlx1, Zeb2 and Tfap2a antibodies (see Materials and Methods). In the notochord, we detected the presence of Dlx1 protein but not Zeb2 or Tfap2a within the *Zeb2#e2* region (Fig. 5). In the mid/hindbrain, these proteins were not detected, while in the forebrain, we detected the presence of all three proteins within the *Zeb2#e2* region. In these experiments, we used DNA sequences that do not contain predicted binding sites for Dlx1, Zeb2 and Tfap2a, as negative controls (Supplementary Material, Table S3). Our results suggest that the Dlx1 protein, which might increase enhancer activity, is found on the *ZEB2#e2* sequence in the notochord, while Zeb2 and Tfap2 proteins that might reduce enhancer activity are not found on this sequence in the notochord. Remarkably, Zeb2 and Tfap2 are present on *ZEB2#e2* sequence in the mid/hindbrain and forebrain, where this *ZEB2#e2* enhancer is not active. Thus, Dlx1, Zeb2 and Tfap2 might play *in vivo* roles in the transcriptional regulation of Zeb2 during brain development.

## Discussion

In this study, we characterized transcriptional enhancers in the *ZEB2* locus that regulate spatiotemporal expression during neurodevelopment. We initially defined the region that most likely contains *ZEB2* regulatory elements in neuronal cells (chr2:145,132,000-145,288,000). The enriched chromatin interactions of the *ZEB2* promoter region with the *ZEB2* TAD region in human fetal brain clearly demonstrated that the *ZEB2* TAD boundary is a central regulatory element that is highly correlated with *ZEB2* expression. Furthermore, the TAD boundary near *ZEB2* is absent in human stem cells and shows differential activity in the germinal zone comprising proliferating neurons, as compared to the cortical plate that is composed of differentiating and migrating neurons, indicating that this TAD boundary is a key element in the regulatory network that controls *ZEB2* expression. Recently, it was shown that genes located in close proximity to the neural-specific TAD boundaries tend to be upregulated, while genes located close to ES-specific borders are less active (22).

Indeed, we identified 18 enhancer candidates from ChIP-seq data sets of human neuronal tissues and MGE progenitors and demonstrated 9 of them to be active enhancers (8 neuronal enhancers and 1 somitic muscle enhancer). Interestingly, the active enhancers were located in the proximal region of the *ZEB2* TAD, suggesting that chromatin interactions, along with epigenetic marks and the TF repertoire, are required for the activity of these enhancers. Each of the eight neuronal active enhancers showed a discrete activity pattern that recapitulated aspects of *ZEB2* expression and which together might play a role in the *ZEB2* regulatory network. These enhancers also presented overlapping activity patterns that likely ensure robust expression of the regulated gene. For example, the enhancers *ZEB2#e2*, *4* and *12* share an overlapping activity pattern that might be executed via different regulatory mechanisms. In addition, not all three are evolutionarily conserved sequences. While the minimal *ZEB2#e2* and *4* sequences are conserved to vertebrates, *ZEB2#e12* is specific to mammals, suggesting that each enhancer has evolved to possess its own function. It also suggests that higher hierarchical organisms require sophisticated and strict regulation of *ZEB2* in order to execute the multifunctionality of this TF.

As transcription factors are required for enhancer actions, we sought the transcription factors that are required for *ZEB2* enhancer activity. We showed that DLX1/2 induced *ZEB2#e2* activity and that deletions of DLX-binding sites reduced (∆169-171, ∆179-183) or totally eliminated (∆39-59) enhancer activity (Fig. 4A and B). As the ∆39-59 mutant lacks the two DLX-binding sites comprising residues 39-42 and 53-57, we further tested the effects of DLX1/2 on each binding site separately and found that loss of either binding site did not disrupt enhancer activity, suggesting that the two sites serve a redundant function (Supplementary Material, Fig. S6D). Dlx1 and Dlx2 are necessary for subpallial development, including interneuron migration to the cortex (11,12,41). Dlx1/2 positively regulates *Zeb2* expression in the subpallium and function upstream of *Zeb2* (10,13). We suggest that Dlx1 protein is presented on *Zeb2#e2* and might increase its activity in the notochord. In addition, AP1, a pioneer factor that is highly expressed in various cell types and found to regulate *ZEB2* expression, was also shown to activate this enhancer (Supplementary Material, Fig. S6B) (16). Furthermore, ZEB2 and TFAP2 proteins that were found to reduce *ZEB2#e2* activity are present in the forebrain but not in the notochord, which suggests that these proteins might repress this enhancer activity in the forebrain but not in the notochord. It also suggests that ZEB2 might have an autoregulatory activity on this specific enhancer. Therefore, DLX1/2 and ZEB2 are thought to have antagonist effects on enhancer activity that could depend on Smad proteins that are known to act with both DLX1/2 and ZEB2 in regulating transcription (1,10). Further investigation is required to identify additional TFs that play a role in *ZEB2* transcription regulation.

Using CRISPR/Cas9 gene editing, we showed that heterozygous deletion of *ZEB2#e2/3* in HEK293 cells significantly reduced *ZEB2* expression (Fig. 4D). Furthermore, the deleted sequence contains two enhancer elements that drove notochord, mid/hindbrain and spinal cord expression in zebrafish and mouse enhancer assays, suggesting that disruption of *ZEB2* enhancers could lead to loss of function of ZEB2. Nucleotide variation could also lead to loss or gain of function of these enhancers. Indeed, we found common SNPs in these enhancers, which suggest that the enhancers are rapidly evolved. However, we found that the characterized TF-binding motifs in *ZEB2#e2* do not contain common SNPs or nucleotide variants (except for a redundant DLX-binding site), suggesting that these TF motifs in *ZEB2#e2* might be under negative selection. In humans, heterozygous mutations or deletions of *ZEB2* have been shown to cause MWS. MWS is characterized by a distinctive facial appearance, intellectual disability and other variable features, including seizures (7). Given the critical role *of ZEB2* in cortical interneuron development, it is possible that disruption of *ZEB2* regulation causes a similar phenotype as does a protein-coding mutation. Indeed, duplication of the *ZEB2* TAD was reported in an MWS patient (42). This duplication contains the *ZEB2* regulatory region and the identified neuronal enhancers that might alter *ZEB2* expression (42). This present study suggests that MWS patients with no *ZEB2*-coding mutations should be screened for mutations in *ZEB2* enhancers to determine the molecular basis of their phenotype.

## Materials and Methods

### ChIP-seq and data set analysis

ChIP-seq data sets of epigenetic marks associated with active enhancers were analyzed to identify enhancer sequences in H9-ESC that were differentiated into inhibitory interneurons (Eshel *et al*., unpublished). At day 26 of culture, human MGE progenitors (1 − 2 × 10^5^) were cross-linked using formaldehyde, lyzed with sodium dodecyl sulfate-based reagents, and chromatin was sonicated on a Bioruptor (Diagenode, Denville, NJ, USA) using a modified ChIP-seq protocol (43). ChIP was performed using antibodies against H3K27ac (Abcam Ab4729) and H3K27me3 (Abcam Ab4729). Prepared libraries from ChIP and input DNA were sequenced on an Illumina HiSeq instrument. For all experiments, reads were mapped to hg19 using BWA (44) and peaks were called using MACS (45). The generated ChIP-seq data will be available in a coming manuscript (Eshel *et al.*, unpublished).

### ChIP-qPCR

Mouse tissues were micro-dissected from E11.5 mice, washed twice in cold phosphate-buffered saline and cross-linked with 1% formaldehyde for 10 min. Chromatin was isolated and sheared using a Bioruptor (Diagenode), and immunoprecipitation was performed using 5 μg of anti-Dlx1 (SAB1412019, Sigma-Aldrich, anti-Tfap2α (sc-12726, Santa Cruz Biotechnology, Dallas, Texas, USA) or anti-Zeb2 antibodies (sc-271984, Santa Cruz Biotechnology, Dallas, Texas, USA). qPCR was performed on targeted sequences using Syber-fast mix (Kapa Biosystems, Roche, Basal, Switzerland) and showed specific enrichment for *Zeb2* enhancers but not for random selected sequences (Supplementary Material, Table S3). Chromatin from the same sample was processed as the input control.

### Transgenic enhancer assays

Primers were designed to amplify candidate enhancer sequences from human and mouse genomic DNA (Supplementary Material, Table S3). PCR products were cloned into the E1b-GFP-Tol2 enhancer assay vector containing an E1b minimal promoter followed by the GFP gene. These constructs were injected into zebrafish embryos using standard procedures. For statistical significance, at least 100 embryos were injected per construct in at least 2 different injection experiments along with Tol2 mRNA to facilitate genomic integration. GFP expression was observed and annotated at 24, 48 and 72 hpf. An enhancer was considered as positive when 30% of the live embryos showed similar and consistence GFP expression patterns. The annotation of the GFP expression driven by the tested minimal enhancer sequences was compared to the GFP-expressed pattern, but not the GFP intensity that was driven by the entire ChIP-seq sequence (the latter not being a quantitative assay). GFP expression was annotated using a SteREO Discovery.V12 fluorescence stereomicroscope (Zeiss).

### Cell culture and reporter assays

A total of 1 – 2 × 10^4^ HEK293 cells were cultured in 96-well plates using standard protocols (46). Cells were transfected with 125 ng of the pGL4.23 plasmid cloned with the enhancer candidate, along with 3 ng of Renilla, using the PolyJet transfection reagent (SignaGen). After 24 h, enhancer activity was measured by the dual luciferase reporter assay (Promega) on a SPARK microplate reader (Tecan). In HEK293 cells, enhancer vectors were co-transfected with 25 ng of the expression constructs pCAG–ZEB2–GFP (a gift from Ruth Ashery-Padan) (47), SP(RSV)-TFAP2α (a gift from Williams Trevor) (48) and CMX-TFAP2γ (a gift from Hubert Schorle) (37) or pß-act-DLX1/2 (a gift from Shigeo Okabe) (49) to measure the effect of each construct on enhancer activity. It should be noted that TFAP2α and TFAP2γ each had an effect on enhancer activity but when introduced together caused the effect to be significantly higher.

### Deletion of *ZEB2#e2/3* sequence in HEK293 cells using the CRISPR/Cas9 system

Heterozygous HEK293 cells deleted of the endogenous *ZEB2#e2/3* allele were created using a CRISPR/Cas9 protocol (50). Briefly, two sgRNAs targeting a 961 bp sequence that encompasses *ZEB2#e2* and *3* (chr2:145188149-145189110; hg19) were designed using CHOPCHOP (51). No potential off-targets were found in searching for matches in the human genome (hg19) when allowing for up to two mismatches in the 20 nt long sequence preceding the protospacer adjacent motif (PAM) sequence. This guide was cloned into pCas9 (BB)2A-GFP (Addgene PX458), as described previously (50). A total of 5 × 10^5^ HEK293 cells were transfected with 5 μg of pX458 [pSpCas9(BB)-2A-GFPsgRNA] using a Lipofectamine 3000 kit (Invitrogen, Carlsbad, California, USA) following the manufacturer’s instructions. Single cells were plated in a 96-well dish for 2 weeks, and then DNA was extracted using Epicentre QuickExtract (QE09050; Lucigen Corporation, Middelton, Wisconsin, USA). PCR amplification and DNA sequencing were performed using the following primers located outside the deleted region: F primer, CAAGGGCTCAATGGAAAGAA; R primer, TGGACCACACAGCTAGAGCA (chr2: 145187215-145189280) to confirm the presence or absence of amplifiable DNA, as compared to wild-type control. Single clone mutant cells were verified for the deletion by Sanger sequencing.

## Supplementary Material

ddy440_Supplementary

ddy440_S1

ddy440_S2

ddy440_S3

ddy440_S4

ddy440_S5

ddy440_S6

ddy440_S7.pdf

ddy440_HMG_Table_S1

ddy440_HMG_Table_S2

ddy440_HMG_Table_S3

ddy440_HMG_Table_S4
