# Supplementary material for: Functional characterization of the ZEB2 regulatory landscape
Source: Hum Mol Genet. 2018 Dec 26;28(9):1487–97. doi: 10.1093/hmg/ddy440 (PMC6466108; doi:10.1093/hmg/ddy440)
Supplement: ddy440_Supplementary [file ddy440_supplementary.docx]

**Supplementary Table 1: List of candidates tested in the zebrafish enhancer assay**

**Supplementary Table 2: Positive enhancers tested in the zebrafish enhancer assay**

**Supplementary Table 3: Primer list**

**Supplementary Table 4: Predicted transcription factor-binding sites for notochord enhancers**

**Supplementary Figure 1: TAD architecture of the *ZEB2* locus**. The heat map represents chromatin interaction frequencies from four different Hi-C datasets. Black arrows represent TAD boundaries of the *ZEB2* locus. **(A)** Hi-C data of H1-ESCs (25). Each square represents two interaction pairs of DNA at 40 kb resolution. **(B)** Hi-C data of human fetal brain from two major zones, the germinal zone (GZ), containing primarily mitotically active neural progenitors, and the cortical and subcortical plate (CP), consisting primarily of post-mitotic neurons (24). Each square represents two interaction pairs of DNA at 10 kb resolution**.** UCSC Genome Browser (http://genome.ucsc.edu) tracks were added (top to bottom) as follows: UCSC genes, positive enhancers in the *ZEB2* locus (from this study), H3K27ac ChIP-seq of H9-derived MGE-like progenitors (Eshel et al, unpublished) and the chrome ImputedHMM track from Roadmap displaying chromatin state segmentation for two different ESCs (H1 and H9), two different ESC-derived neuron progenitors (H1-derived neuron progenitors, H9-derived neurons), H9-derived neurons and five different brain regions (fetal female and male brains, anterior caudate, hippocampus (middle) and inferior temporal lobe).

**Supplementary Figure 2: *ZEB2* expression during hESC differentiation into inhibitory interneurons. (A)** *ZEB2* expression levels in H1-hESCs (day 0), MGE-like progenitors (day 26), inhibitory GABAergic–like interneurons (day 39) and somatostatin-enriched interneurons (day 55). **(B)** *In situ* hybridization of zeb2a in zebrafish embryos at 24 and 48 hpf (adapted from Thisse & Thisse, 2008).

**Supplementary Figure 3: Functional characterization of *ZEB2#e2*, *e3*, *e4* and *e5* enhancers in zebrafish**. Two representative larvae with similar GFP expression patterns for each enhancer at 24, 48, 72 hpf. **(A)** *ZEB2#e2* drove GFP expression in the notochord at 24, 48 and 72 hpf. **(B)** *ZEB2#e3* drove GFP expression in the midbrain, hindbrain and spinal cord at 24 and 48 hpf. **(C)** *ZEB2#e4* drove GFP expression in the notochord at 24, 48 and 72 hpf. **(D)** *ZEB2#e5* drove GFP expression in the central nervous system at 24 hpf and drove GFP expression in the midbrain, hindbrain, spinal cord and somitic muscles at 48 hpf.

**Supplementary Figure 4: Functional characterization of *ZEB2#e6*, *e7*, *e12*, *e13*, and *e14* in zebrafish.** Two representative larvae with similar GFP expression patterns for each enhancer at 24, 48, 72 hpf. **(A, B)** *ZEB2#e6* and *ZEB2#e7* drove GFP expression in bilateral neurons near the eye at 24 and 48 hpf. **(C)** *ZEB2#e12* drove GFP expression in the notochord at 24, 48 and 72 hpf. **(D)** *ZEB2#e13* drove GFP expression in somitic muscles at 48 and 72 hpf. **(E)** *ZEB2#e14* drove GFP expression in the central nervous system at 24 hpf.

**Supplementary Figure 5: Segmental analysis of the *ZEB2#e5* enhancer in zebrafish embryo 48 hpf**. **(A-C)** Segmental analysis of *ZEB2#e5* that was divided into three overlapping segments (segments 1-3). **(A)** UCSC Genome Browser conservation track (http://genome.ucsc.edu). **(B)** A graph displaying the number of embryos showing GFP expression in the spinal cord and mid/hindbrain out of all live embryos at 48 hpf. **(C)** Zebrafish enhancer assays for *ZEB2#e5* segments show spinal cord and mid/hindbrain GFP expression for the entire ChIP-seq peak, as well as for segment 2+3, segment 3 and the zebrafish-conserved segment 3 sequence.

**Supplementary Figure 6: TFs modulate *ZEB2#e2 and ZEB2#e4* activity. (A)** Co-transfection with *TFAP2α* results in a significant increase in activity of the *ZEB2#e4* minimal region. **(B)** Co-transfection with *AP-1* causes a significant increase in *ZEB2#e2* minimal region activity. **(C)** Co-transfection with the *ZEB2#e2*_∆179-183 mutant with *DLX1*/*2* results in a significant reduction in *ZEB2#e2* activity. **(D)** Co-transfection of *ZEB2#e2*_∆39-42 and *ZEB2#e2*_∆53-57 mutants with *DLX1/2* show similar activity as the *ZEB2#e2* minimal region.

**Supplementary Figure 7: ZEB2#e2 nucleotide variants in human populations.** Snapshot of UCSC Genome Browser tracks of the *ZEB2#e2* minimal sequence. The six functional TFBSs are marked by black rectangles. Top panel: No common SNPs (dbSNP150, MAF>1%) are detected (dbSNP150, MAF>1%). Middle panel: All nucleotide variants in this sequences are presented (dbSNP150, MAF<1%), with no nucleotide variants being detected in TFBSs, except for DLX_BS_del 39-42 (rs534830312, MAF=0.002%; rs776586183, MAF=0.00004%; rs545668291, MAF=0.00%). Bottom panel: UCSC Genome Browser conservation track of the *ZEB2#e2* minimal sequence.
