# Supplementary figures and images for: Functional characterization of the ZEB2 regulatory landscape
Source: Hum Mol Genet. 2018 Dec 26;28(9):1487–97. doi: 10.1093/hmg/ddy440 (PMC6466108; doi:10.1093/hmg/ddy440)

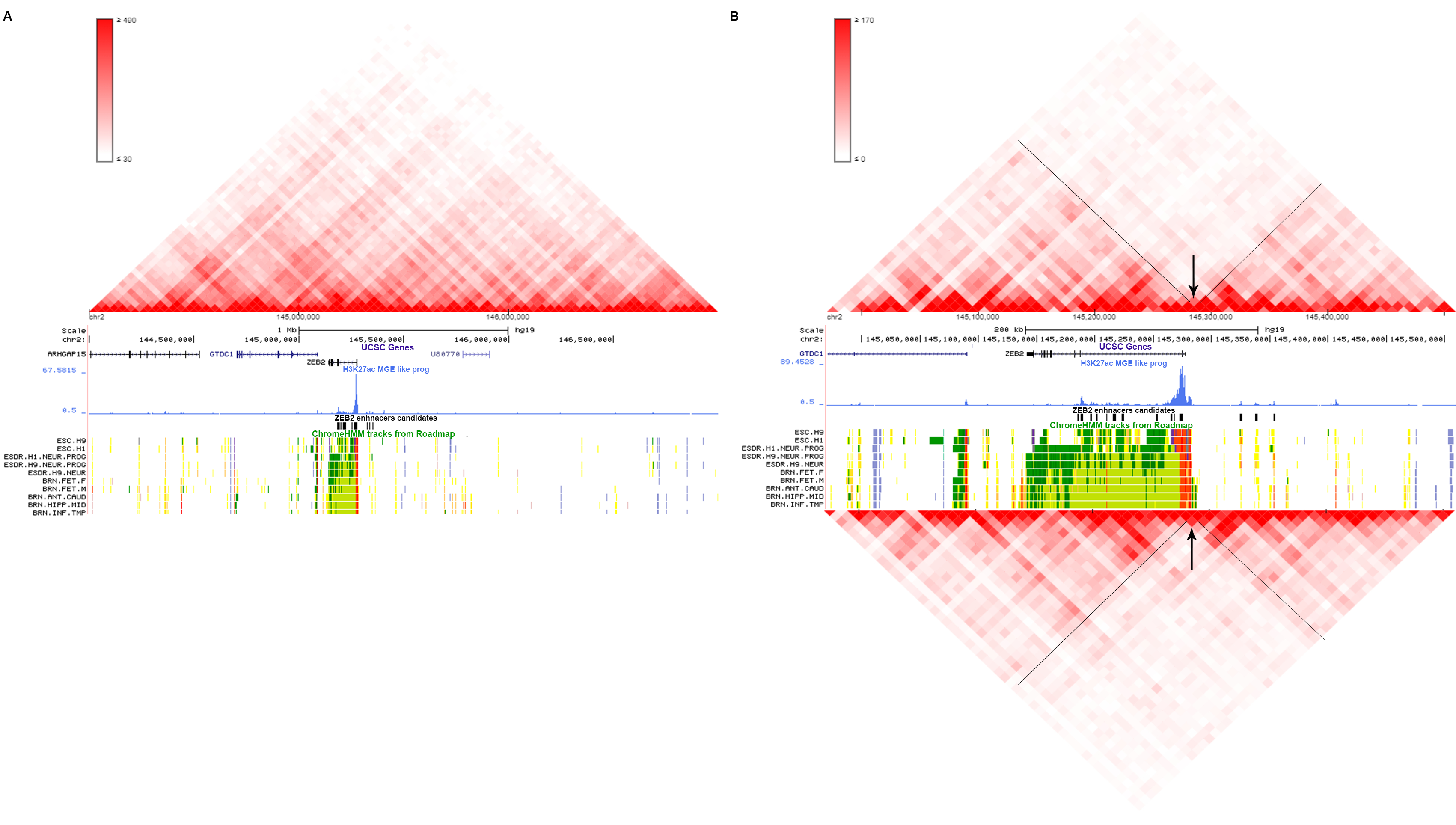

Supplement: ddy440_S1 [file ddy440_s1.png]

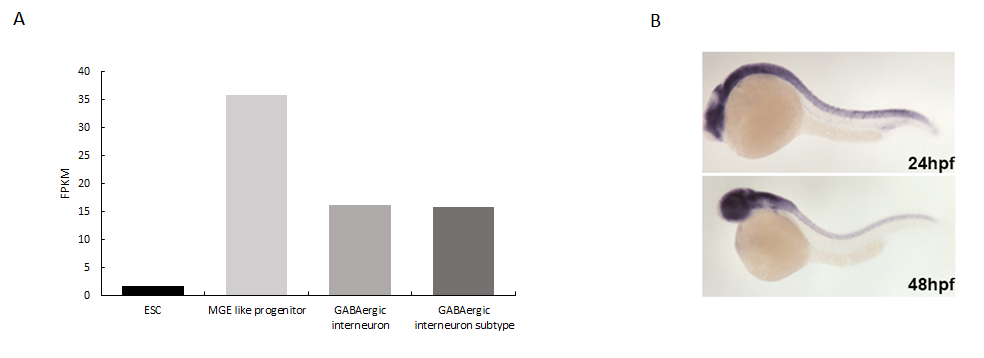

Supplement: ddy440_S2 [file ddy440_s2.png]

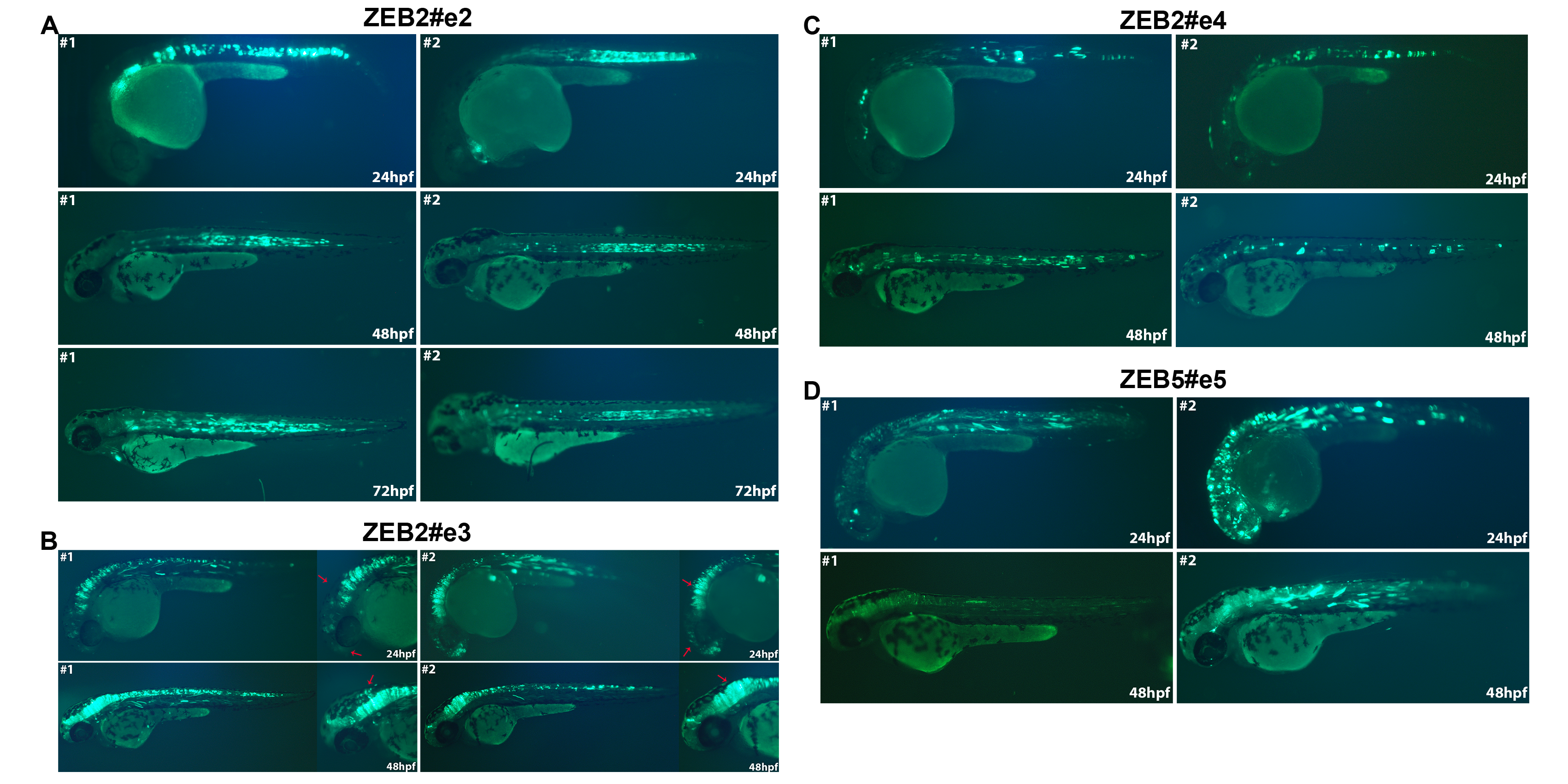

Supplement: ddy440_S3 [file ddy440_s3.png]

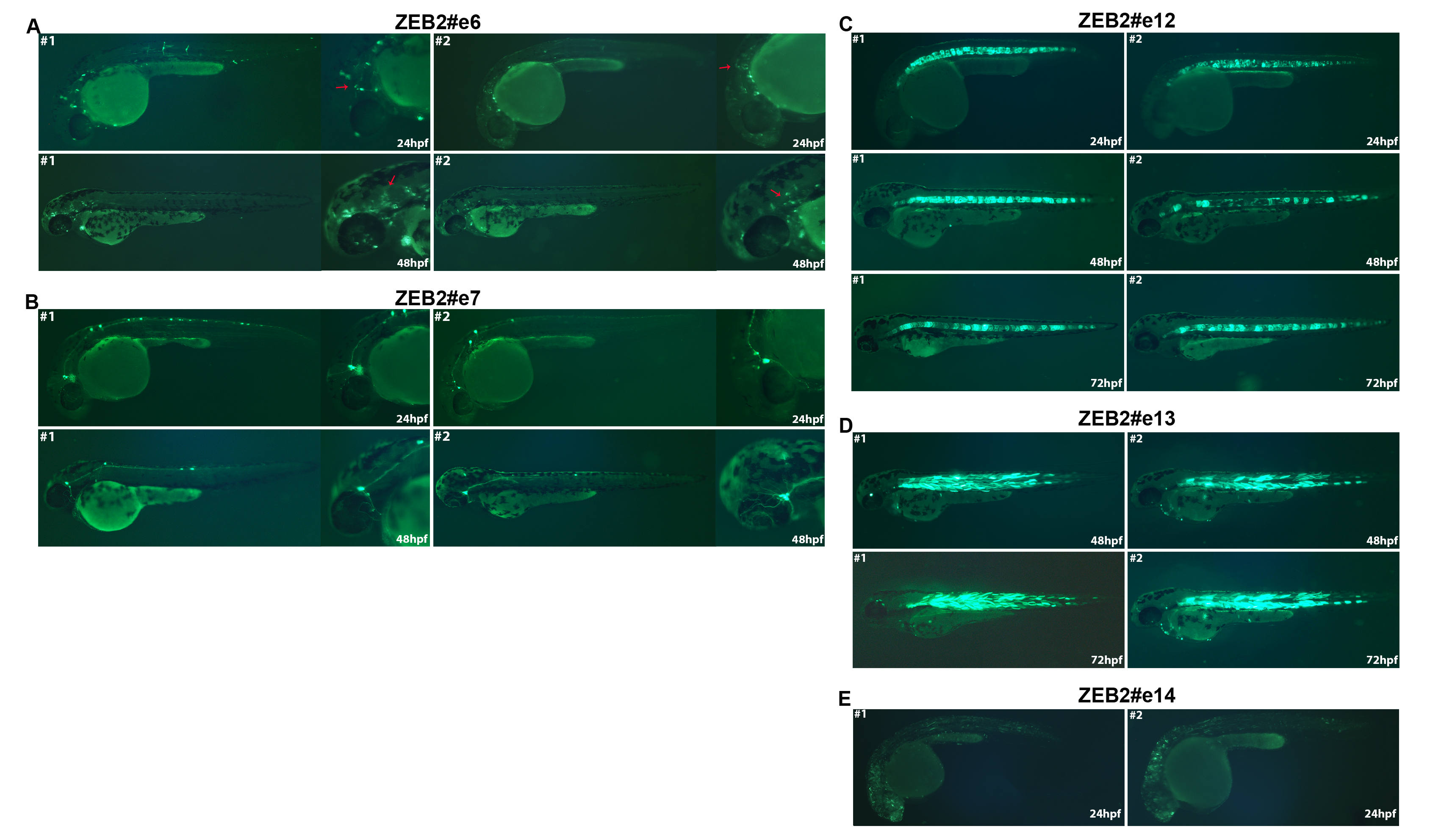

Supplement: ddy440_S4 [file ddy440_s4.png]

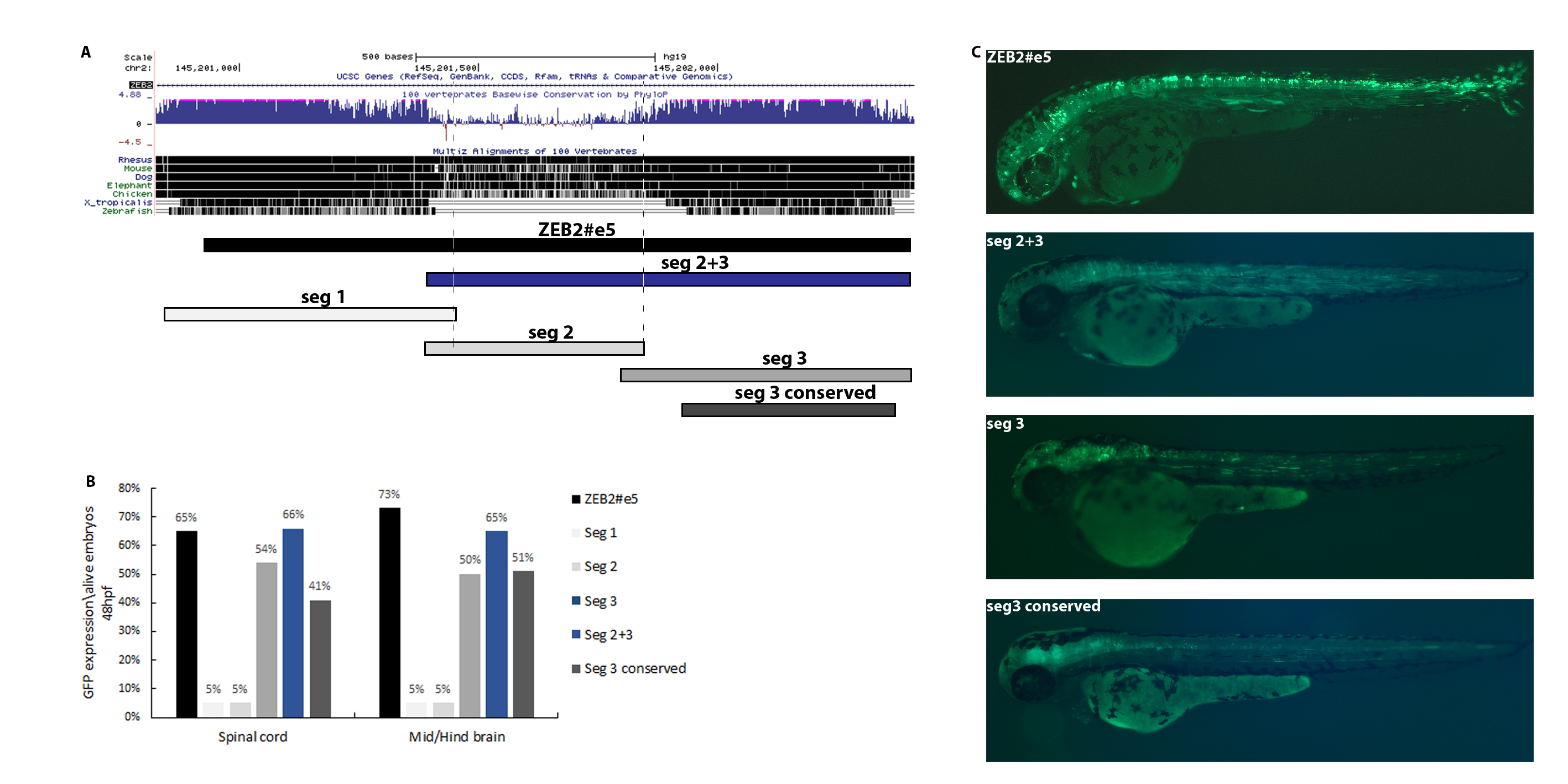

Supplement: ddy440_S5 [file ddy440_s5.png]

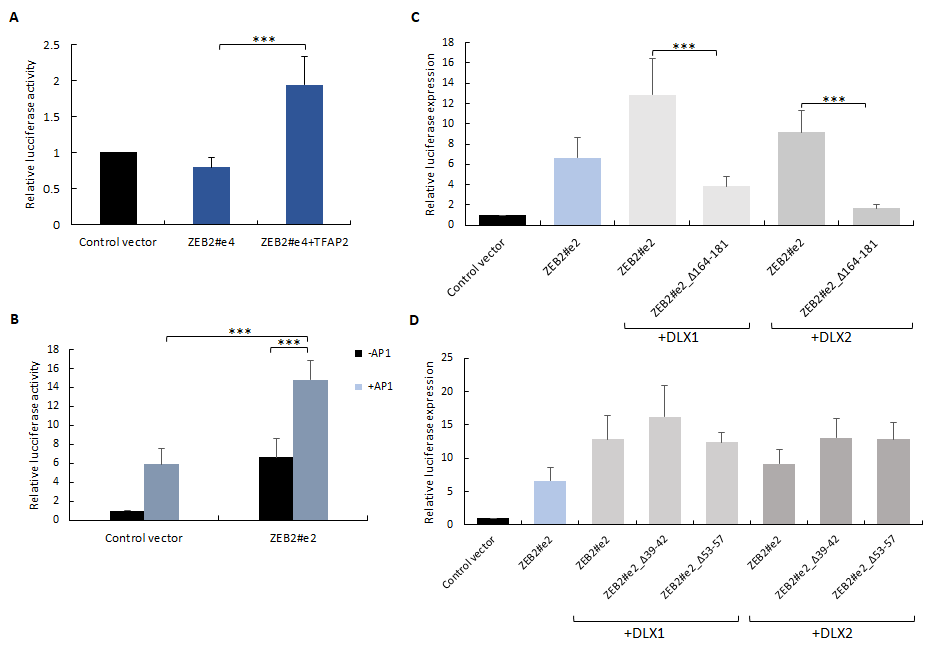

Supplement: ddy440_S6 [file ddy440_s6.png]

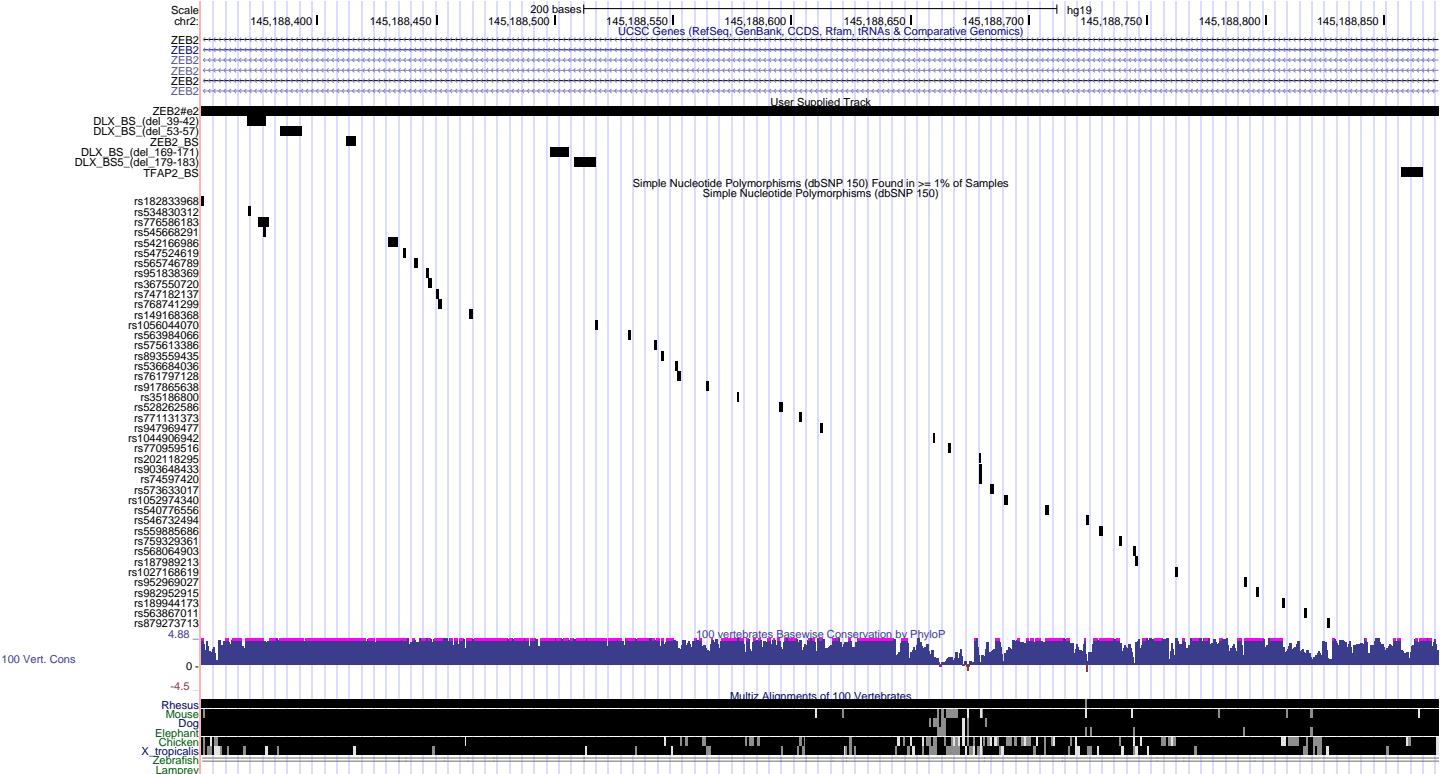

Supplement: ddy440_S7.pdf [file ddy440_s7.pdf]
